# Assessing the Quality of Simulated Food Patterns with Reduced Animal Protein Using Meal Data from NHANES 2017–2018

**DOI:** 10.3390/nu15112572

**Published:** 2023-05-31

**Authors:** Maria F. Vasiloglou, Paloma Elortegui Pascual, Eric A. Scuccimarra, Roko Plestina, Fabio Mainardi, Tsz-Ning Mak, Frédéric Ronga, Adam Drewnowski

**Affiliations:** 1 Nestlé Institute of Health Sciences, Nestlé Research, 1000 Lausanne, Switzerland; paloma.elorteguipascual@rd.nestle.com (P.E.P.); ericantoine.scuccimarra@rd.nestle.com (E.A.S.); roko.plestina@rd.nestle.com (R.P.); fabio.mainardi@rd.nestle.com (F.M.); frederic.ronga@rd.nestle.com (F.R.); 2 Nestlé Institute of Health Science Singapore Hub, 29 Quality Road, Singapore 618802, Singapore; tszning.mak1@rd.nestle.com; 3 Center for Public Health Nutrition, University of Washington, Seattle, WA 98195, USA; adrewnow@fredhutch.org

**Keywords:** simulation, modeling, plant-based, flexitarian, vegetarian, HEI

## Abstract

The nutritional consequences of progressively replacing meat products with plant-based foods need to be systematically evaluated. Modeling analyses provide insights into the predicted food consumption and nutritional adequacy of plant-based diets. We developed a novel methodology to simulate food patterns and evaluate diet quality. Meal data from the National Health and Nutrition Examination Survey (NHANES) 2017–2018 was used to create 100 7-day meal plans subject to various nutrient and food group optimization criteria. Omnivore (reference diet), flexitarian, pescatarian, and vegetarian food patterns were modeled using mixed integer linear programming. The modeled food patterns used the 25th and 75th percentiles of the US Usual Dietary Intakes to set the optimization constraints. The diet quality was determined using the Healthy Eating Index 2015 (HEI-2015). The modeled vegetarian, pescatarian, and flexitarian food patterns outperformed the omnivore diet on the HEI-2015, with the vegetarian pattern achieving the highest score (82 for females, 78 for males). Modeled flexitarian patterns, with a 25 to 75% reduction in animal protein, offer viable options for those seeking to reduce but not eliminate their animal protein intake while supporting the transition from omnivore to fully plant-based diets. This methodology could be applied to evaluate the nutrient and diet quality of different dietary patterns with various constraints.

## 1. Introduction

The current shift toward plant-based foods in Western economies has been accelerated by health, environmental, and ethical concerns [1]. Plant-based diets restrict meat consumption and are often built around whole grains, fruits, vegetables, nuts, and legumes, which are good sources of unsaturated fats; dietary fibers; and some vitamins, minerals, and phytonutrients [2,3,4]. Dietary patterns based on nutrient-dense plant foods [2] have been associated with a lower risk of non-communicable diseases (NCDs), including cardiovascular diseases [5,6,7], metabolic syndrome [7], type 2 diabetes [7,8], and some cancers [9]. Another example of a nutrient-dense plant-based pattern is the Mediterranean diet [10]. However, exclusively plant-based diets can be low in iron, zinc, iodine, calcium, vitamin D, and vitamin B12 [11].

Systematic assessments of the nutritional value of alternative dietary patterns that limit meat consumption remain limited [2]. Relatively few studies have evaluated the nutrient density of the “flexitarian” diet, which remains poorly defined [3,5,6,7,8,9]. The terms flexitarian or semi-vegetarian have been used to describe consumers who their limit consumption of certain types of meat, such as red meat, poultry, or fish, either in terms of frequency or portion size [12]. A survey conducted in the USA in 2018 found that one in three Americans consider themselves “flexitarians” [13]. Although there is a clear significant movement of consumers adopting more plant-centric diets, 85% of the US population is still eating meat and meat products [14]. Pescatarians consume fish as opposed to red meat and poultry. Vegetarians or lacto-ovo-vegetarians avoid any type of animal flesh but do consume eggs and dairy (milk, yogurt, and cheese). Vegans, the least numerous group, avoid all animal products and exclusively consume foods that are derived from plants.

The nutritional consequences of progressively replacing meat products with plant-based foods need to be systematically evaluated. In this context, modeling analyses can provide some insight into the predicted nutrient intakes and nutritional adequacy of flexitarian, pescatarian, and vegetarian dietary patterns.

In this study, we modeled alternative food patterns using a diet optimization model based on mixed integer linear programming (MILP), which is a variant of linear programming where some of the variables are constrained to be integers while some others are allowed to be non-integers [15]. Our goal was to model food patterns by generating meal plans that adhered to specific dietary criteria (e.g., flexitarian, pescatarian, and vegetarian). For this purpose, we used a test case that consisted of simulating different degrees of reduced animal protein food patterns in US adults aged 31–50 years old. Our secondary objective was to assess the diet quality between modeled food patterns diets using the Healthy Eating Index (HEI-2015).

## 2. Materials and Methods

### 2.1. Defining Food Patterns

Meals consumed by adult NHANES participants were used to create modeled alternative food patterns following the rules described in Table 1. The meals were used as reported with no changes. The US reference (omnivore) pattern was defined as being based on meals that included foods of both animal and plant origin. No exclusion criteria were applied. Defining the flexitarian pattern was difficult, as a formal definition or evidence base is currently lacking. We defined flexitarian food patterns as being progressively more restricted in meals containing animal-sourced foods (meat, organ meat, poultry, seafood, eggs, and dairy). The present a priori cut points were a 25% reduction, a 50% reduction, and a 75% reduction relative to the US reference habitual diet. It should be noted that the meal inclusion criteria can overlap, e.g., a vegetarian meal is also an omnivore meal, and any omnivore meal can fit the flexitarian food pattern. The vegetarian (ovo-lacto-vegetarian) pattern was defined as excluding meals that contained animal flesh (meat, organ meat, poultry, or seafood). The pescatarian pattern was defined as excluding meals that contained meat, organ meat, and poultry, but allowing seafood.

### 2.2. Food Pattern Simulation Using Mixed Integer Linear Programming (MILP)

**Figure 1:**
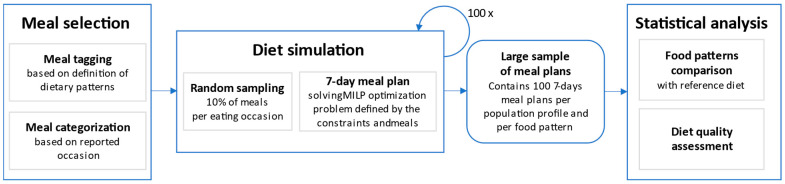
Schematic overview of the generation of 7-day meal plans to simulate food patterns.

Assessing the overall nutritional value of alternative food patterns can be a challenge, given that the number of recalled meals fitting the proposed criteria can be small, even in the large NHANES 2017–2018 database [16]. Therefore, we adopted a food pattern modeling approach by using meals reported by NHANES adult participants to create a large number of alternative food patterns. Mixed integer linear programming (MILP) was used to create optimized US reference, vegetarian, pescatarian, and 3 varieties of flexitarian food patterns, as defined in Table 1. In MILP modeling, optimized food patterns are mathematically represented using an objective function of continuous variables to be determined and subjected to linear equality and inequality constraints (e.g., nutrient requirements or distribution of food groups). Since some of the food group constraints were formulated by the USDA on a weekly basis (e.g., cup eq per week), a 7-day meal plan was the most appropriate model that would allow all dietary consumption constraints to be fully applied. An overview of the modeling approach is shown in Figure 1.

In the present study, MILP was used to generate 100 individual 7-day meal plans for each of the five dietary patterns and the two age–sex groups by optimally assigning meals retrieved from NHANES to specific days. The use of MILP rather than LP permitted the creation of variables that could only take integer values. For example, in this study we defined a variable that took a value of 0 or 1 for each meal, indicating whether the meal was included in the diet or not on a specific day and eating occasion. The simulation of one diet (100 7-day meal plans) took less than 2 h on a reasonably powerful desktop computer. The weekly averages of the 100 meal plans per modeled food pattern and per sex can be found in the Supplementary Materials.

### 2.3. Study Population and Habitual Intakes by Age-Sex Group

For the present study, the dietary intake data came from the latest cycle of the nationally representative US National Health and Nutrition Examination Survey (NHANES) 2017–2018 [16], using 28,802 meals reported by 5856 adult participants aged 18 to 80. Energy and nutrient composition data for all foods and beverages came from the USDA Food and Nutrients Database for Dietary Studies (FNDDS) [17], and portion sizes and recipes were used to calculate the energy and nutrient contents of meals [16]. The USDA Food Patterns Equivalents Database (FPED) 2017–2018 [18] was used to convert foods and beverages consumed by NHANES participants into 37 USDA food pattern components. In order to monitor whether Americans meet the food pattern recommendations of the Dietary Guidelines of Americans (DGA) [19], the foods in the FNDDS must be converted to the respective amounts of food pattern equivalents present in them, expressed in ounces equivalents (oz eq) and cup equivalents (cup eq) [18]. The FPED includes the amounts of 37 food pattern components (fruits, vegetables, grains, protein foods, dairy, oils, added sugars, solid fats, and alcoholic drinks) present in 100 g of each of the FNDDS foods, which is useful for calculating the Healthy Eating Index (HEI), which is a measure of adherence to the DGA [19] (see Appendix A).

To better understand the habitual intakes by age–sex group, we used the most recent (2007–2010) National Cancer Institute (NCI) Usual Dietary Intakes [20], which estimates the distribution of usual intake for a population or subpopulation, expressed in cup equivalents (cup eq) or ounce equivalents (oz eq) [18], and addresses measurement errors associated with 24 h recalls. Published NCI data [20] was used to set consumption constraints for the linear programming (LP) food plan optimization program for adult men and women (31–50 years age range), with energy intakes set at 2400 kcal/day for men and 1800 kcal/day for women [20]. Food consumption percentiles at the 25th percentile and 75th percentile were used to set the optimization constraints in the linear programming model (see Section 2.6).

### 2.4. Input Data from NHANES 2017–2018 Meals

A large set of realistic meals was required to create alternative meal plans that were sufficiently diverse to allow for optimization. Rather than attempting to artificially construct a dataset of meals, 28,802 adult meals reported in NHANES in 2017–2018 served as input for the MILP algorithm.

First, the observed meals were classified into 5 different food patterns (see Table 2). The meals were also categorized by the reported eating occasion. Breakfast meals were those reported as “breakfast” or “desayuno”; lunch meals were those reported as “lunch”, “almuerzo”, or “comida”; dinner meals were those reported as “dinner”, “supper”, and “cena”; and snacks were eating events reported as “snack”. The result of the two classifications is shown in Table 2. The vegan food pattern was not attempted in the MILP analyses due to the scarcity of vegan meals (*n* = 1842; of which 760 were snacks).

The observed meals in different categories were the input data for the MILP algorithm that generated a 7-day meal plan for each of the alternative food patterns and the two age–sex groups. A 10% sample of the available NHANES meals was sampled without replacement to create each 7-day meal plan. This method was used to ensure that the generated optimized 7-day meal plans were diverse and that the selected number of meals was large enough to allow for flexibility in the optimization.

### 2.5. Setting Optimization Constraints

In the MILP model, the objective function was created to minimize the deviation of the 7-day meal plans from the desired ranges for nutrient and USDA food pattern components, which we refer to as “optimization constraints”. To create 7-day meal plans that were as close as possible to the US reference diet, the food groups and USDA food pattern components were constrained to a range defined by the 25th and 75th percentiles of the NCI usual intakes [20]. The USDA food pattern components from FPEDs [21] that were consumed daily were set as daily constraints, while food pattern components that were consumed episodically were set as weekly constraints. The optimal ranges for nutrients and USDA food pattern components for the US reference food pattern (omnivore) are listed in Table 3.

For the vegetarian modeled food pattern, the upper limits were increased by 100% for the total protein from legumes and total soy, nuts, and seeds food patterns to compensate for the animal protein, which was decreased by 100%; the upper limits were decreased by a factor of two for solid fats; finally, the amounts of meat, poultry, seafood, and eggs were set to the usual intakes of eggs, which was the only component allowed in the vegetarian diet.

For the modeled pescatarian food pattern, the rules were the same as for the modeled vegetarian food pattern with total meat, poultry, seafood, and eggs set to the usual intakes for seafood plus eggs. A separate rule set the range for seafood to be equal to the usual intakes of seafood in the NCI database [20].

For the flexitarian modeled food pattern, the omnivore optimization constraints (Table 3) were adjusted to limit the proportion of animal-sourced foods, mostly meat. For animal protein foods, the upper limit was progressively reduced to yield the 75%, 50%, and 25% animal protein flexitarian modeled food patterns, and the lower limit was decreased to maintain the same ranges as in the omnivore pattern. For plant protein foods, both the upper and lower limits were increased by a factor of 1.75, 1.5, or 1.25 in the 25%, 50%, or 75% flexitarian modeled food patterns, respectively, to compensate for the reduction in animal protein. The optimization constraints for modeled Flexi75, Flexi50, Flex25, pescatarian, and vegetarian diets are shown in Appendix A.2 (Table A2, Table A3, Table A4, Table A5 and Table A6).

### 2.6. The MILP Algorithm for the Generation of 7-Day Meal Plans

The mathematical techniques used are described as follows. Let *n* be the number of randomly selected meals and *m* the number of food pattern components considered in the constraints. Each day was simulated by minimizing the diet’s objective function. Instead of using “hard constraints”, to ensure that the solution strictly fulfilled the optimization constraints, “soft constraints” or “slack variables” were used to ensure a solution could always be found:

(1) $$minimize\left(\sum_{j=1}^{m}\left(W_{j}^{L}s_{j}^{L}+W_{j}^{U}s_{j}^{U}\right)\right)$$

subject to

(2) $$C_{j}^{L}-s_{j}^{L}\leq \sum_{i=1}^{n}F_{ij}x_{i}\leq C_{j}^{U}+s_{j}^{U},\;\mathrm{for}\;j=1\;to\;m;$$

(3) $$x_{i}\in \left\{0,1\right\},\;\mathrm{for}\;i=1\;to\;n;$$

(4) $$\sum_{i=1}^{n}T_{ik}x_{i}=1,\;\mathrm{for}\;k=1\;to\;4;$$

where the index *i* represents the candidate meals; the index *j* is a meal’s food component or nutrient (e.g., energy, protein, or sodium); the index *k* represents the eating occasion (i.e., breakfast, lunch, snack, or dinner); $x_{i}$ is the integer variable indicating whether the *i*th meal was used in the daily meal plan; $F_{ij}$ is the amount of food component or nutrient *j* in the *i*th meal; $C_{j}^{L,U}$ represents the lower (L) and upper (U) bounds of the desired target range for the *j*th meal attribute; $s_{j}^{L,U}$ are the lower and upper slack variables for the *j*th meal attribute; $W_{j}^{L,U}$ indicates the weight of the corresponding slack variable in the objective function; $T_{ik}$ are constants with values 0 or 1 indicating whether the *i*th meal can be used as the *k*th eating occasion.

In the above MILP model, the objective function (Equation (1)) was created to minimize the deviation of the diets from the desired nutrient and food group ranges. Equation (2) expresses the soft optimization constraints using the slack variables $s_{j}^{L,U}$ that provide flexibility for the created meal plan to fall outside of the target ranges. Each slack variable was paired with a weight $W_{j}^{L,U}$ that controlled the relative importance of each target and allowed for adjustments to the allowed deviations from the target ranges (e.g., for non-meat food components in the non-omnivore diets). When the objective function was minimized, the slack variables were also minimized, resulting in a meal plan containing the combination of meals that best fitted within the desired nutritional ranges, while allowing for some deviation if required. For example, a simulated female vegetarian food pattern could have 1.448 cup-equivalents of fruit over the course of a day. This is over the 75% usual intake for females of 1.3 cup. In this case, the slack variable was equal to 1.448−1.3=0.148 cups.

Equation (3) means that the *i*th meal can be either used as reported in the NHANES or not used at all. With Equation (4), the solution is forced to contain exactly one each of breakfast, lunch, dinner, and snack per day. To further ensure the diversity of the modeled patterns, we converted the weekly constraints to daily constraints, making sure they summed up at the weekly level and allowed meals to be reused only after a predefined number of days (breakfast every day, lunch after 5 days, dinner after 7 days, and snack after 3 days).

### 2.7. Validation of the MILP Approach

**Figure 2:**
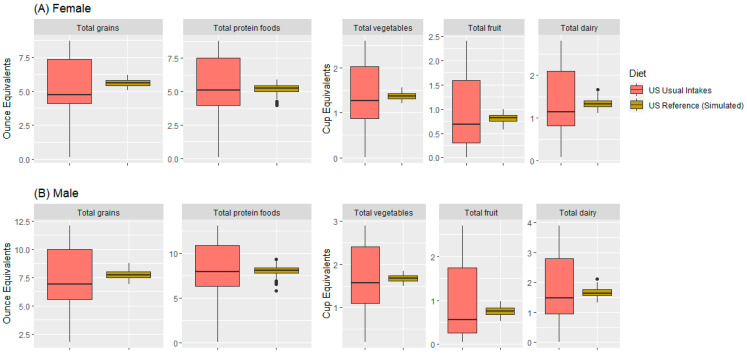
Distribution of the simulated intakes (dark yellow) compared with usual intakes (light red) of the main FPEDs for female (**A**) and male (**B**) NHANES 2017–2018 participants. Dots represent the outliers.

The modeled US reference food pattern (omnivore) was compared with the distributions of usual intakes of the USDA major food groups, as captured in the NCI usual dietary intakes of main US dietary patterns (i.e., Healthy U.S.-Style and Healthy Vegetarian and Healthy Mediterranean-Style) [20]. Figure 2A shows box plots by food group for women. Figure 2B shows boxplots by food group for men. The modeled US reference food pattern tracked the usual intakes of the main food pattern components. However, the variance in the modeled patterns was significantly smaller, as expected from (1) the fact that the modeled patterns were an average of 7 days, whereas the usual intakes were for a single day, and (2) the number of constraints forced the modeled patterns toward the mean.

### 2.8. Diet Quality of the Modeled Patterns

The Healthy Eating Index (HEI) is a 100-point measure of diet quality based on the degree of compliance with the DGA [22]. The most recent HEI-2015 is made up of 13 sub-scores that include both nutrients and food pattern components [22]. The dietary constituents for calculating the HEI-2015 component and total scores are as follows: total fruit, whole fruit (total fruit excluding juice, i.e., citrus, melons, and berries, plus other intact fruits), total vegetables, dark green vegetables, legumes (beans and peas), whole grains, dairy (milk, yogurt, cheese, and fortified soy beverages in the form of skim milk equivalents), total protein foods (lean fraction only), seafood, eggs, soy products, nuts and seeds, refined grains, saturated fatty acids, polyunsaturated fatty acids, monounsaturated fatty acids, sodium, calories from added sugars, and total calories.

### 2.9. Statistical Analysis

For each 7-day meal plan, we computed the daily means and analyzed the distribution of nutrients and food groups. We also tested the dietary variety, which was defined as the number of distinct meals per week. The meal plans contained a mean of 25.9 unique meals per week out of 28 eating occasions, with the more restrictive diets having, on average, fewer unique meals. The variety metric was not intended to measure the diversity of foods consumed but merely to ensure that the same meals did not recur too often in the modeled food patterns. The mean and standard deviation (SD) of nutrient intakes for the modeled US reference (omnivore), flexitarian, and vegetarian food patterns were calculated. Omnivore food patterns were then compared with the alternative food patterns and components that were found to be significantly different were reported. The statistical differences in food patterns between the models were assessed using Wilcoxon tests, which were adjusted for multiplicity using the Benjamini–Hochberg procedure with α=0.05. All of the differences in food patterns reported below were statistically significant. The means and SDs of the total and component scores for the HEI-2015 were calculated for all the modeled food patterns. The total and component scores of the HEI-2015 were compared using ANOVA, and significant differences between the omnivore diet and the other diets were subjected to a post hoc pairwise comparison using the Benjamini–Hochberg procedure with α=0.05 The analysis was performed using Python version 3.8, and R version 4.0.5 was used to produce the plots.

## 3. Results

### 3.1. Modeled Food Patterns

#### 3.1.1. Contribution of Animal Protein (Meat, Poultry, Seafood, and Eggs, Excluding Dairy) in the Modeled Food Patterns

**Figure 3:**
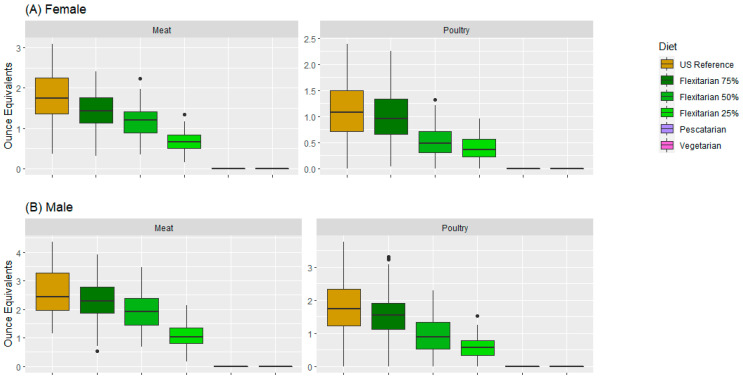
Red meat and poultry amounts (in oz eq) for the modeled US reference, Flexi75, Flexi50, Flexi25, pescatarian, and vegetarian food patterns in the 31–50-year-old female (**A**) and 31–50-year-old male (**B**) data. Dots represent the outliers.

Both meat and poultry amounts had a gradual decrease from the US reference to Flexi25 in both the female and male modeled data. The modeled pescatarian and vegetarian patterns did not contain any amount of meat and poultry, with amounts equal to 0 oz eq. Higher amounts of meat and poultry were generated in the modeled male data (Figure 3B) compared with the modeled female data (Figure 3A) for all modeled food patterns.

**Figure 4:**
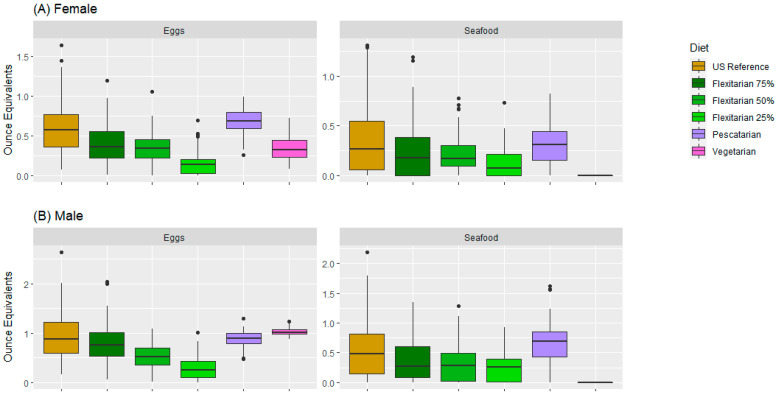
Egg and seafood amounts (in oz eq) for the modeled US reference, Flexi75, Flexi50, Flexi25, pescatarian, and vegetarian food patterns in the 31–50-year-old female (**A**) and a 31–50-year-old male (**B**) data. Dots represent the outliers.

The egg and seafood amounts differed in the female and male modeled data. Egg amounts gradually decreased from the modeled US reference pattern to the modeled Flexi25 pattern in both the female and male diets, meanwhile, they increased in the pescatarian and vegetarian modeled patterns. The egg quantity was significantly lower in the modeled vegetarian and pescatarian female data (Figure 4A) compared with the modeled male data (Figure 4B). The highest egg amounts were found in the modeled vegetarian male data (1.02 oz eq). The seafood amounts were higher in the modeled pescatarian pattern compared with all flexitarian and US reference modeled patterns. The seafood amount was lower in females than in males. There was no seafood in the vegetarian diet (0 oz eq).

#### 3.1.2. Contribution of Dairy in the Modeled Food Patterns

**Figure A1:**
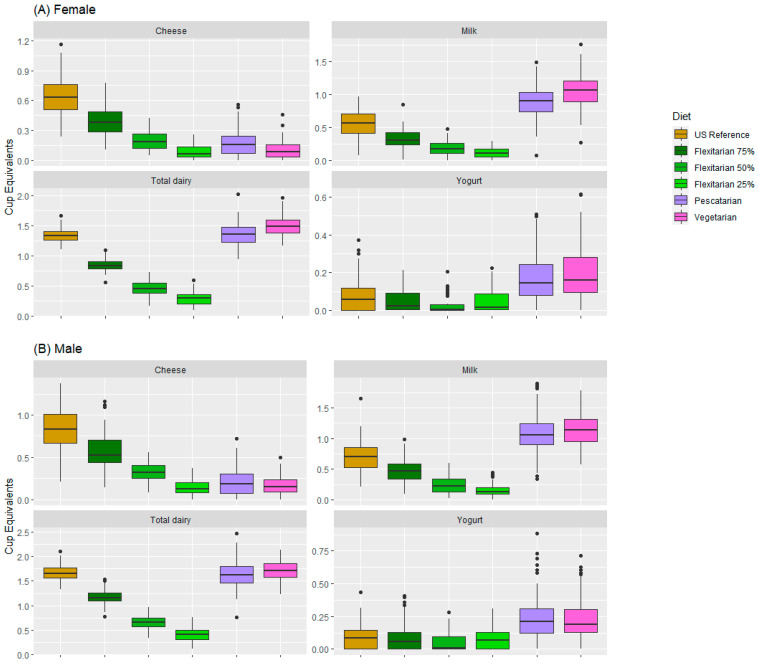
Dairy (cheese, milk, yogurt, and total dairy) amounts (in cup eq) for the modeled US reference, Flexi75, Flexi50, Flexi25, pescatarian, and vegetarian food patterns in the 31–50-year-old female (**A**) and 31–50-year-old male (**B**) data. Dots represent the outliers.

There was a monotonic decrease in cheese, milk, and total dairy amounts in all modeled flexitarian food patterns from the US reference to Flexi25 (Appendix A Figure A1). All dairy food groups (including cheese, milk, yogurt, and total dairy) had comparable amounts in the modeled vegetarian and pescatarian patterns. The milk and total dairy amounts were considerably higher in the modeled vegetarian and pescatarian patterns compared with the modeled flexitarian patterns. Yogurt had similar quantities among the modeled US reference pattern and the modeled flexitarian patterns, while the modeled pescatarian and modeled vegetarian patterns had higher quantities. Lastly, all dairy food groups had comparable amounts in both the female (Appendix A Figure A1A) and male (Appendix A Figure A1B) modeled data.

#### 3.1.3. Contribution of Plant Protein in the Modeled Food Patterns 

**Figure A2:**
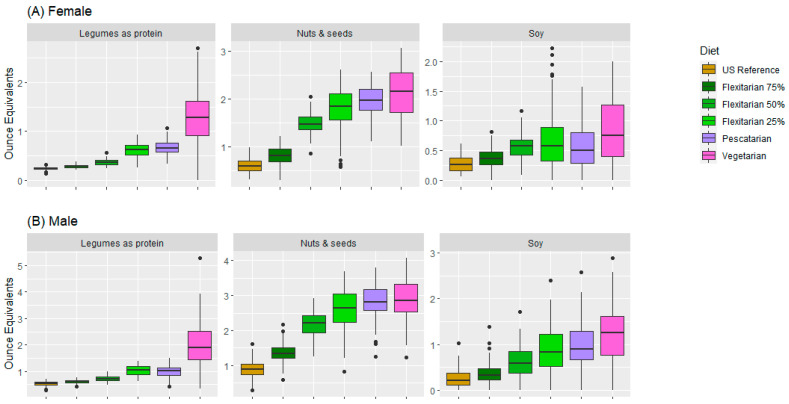
Plant protein food amounts from legumes as proteins, nuts and seeds, and soy sources (in oz eq) for the modeled US reference, Flexi75, Flexi50, Flexi25, pescatarian, and vegetarian food patterns in the 31–50-year-old female (**A**) and 31–50-year-old male (**B**) data. Dots represent the outliers.

There was an overall increase in the legume amounts, as well as nuts and seeds, across all modeled female food patterns from the US reference to vegetarian patterns (Appendix A Figure A2A). A similar increase was observed in the modeled male data (Appendix A Figure A2B). The highest amounts of legumes and nuts and seeds were observed in the vegetarian models for both females and males (2.16 oz eq and 2.87 oz eq, respectively). In addition, legumes as proteins and nuts and seeds amounts were higher for males than for females across all modeled food patterns. The soy amount did not exhibit a significant trend across the modeled female data, but it showed a gradual increase in the modeled male data from the US reference to vegetarian patterns.

#### 3.1.4. Contribution of Total Protein: Animal Protein Food (Excluding Dairy) and Plant Protein in the Modeled Food Patterns

**Figure A3:**
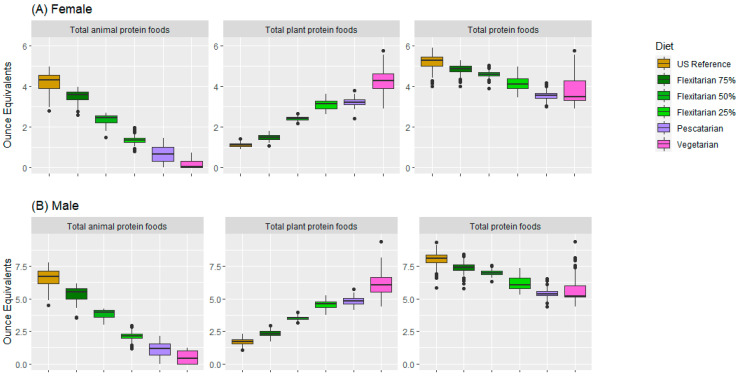
Total protein foods (plant protein sources and animal sources, excluding dairy) content (in oz eq) for the modeled US reference, Flexi75, Flexi50, Flexi25, pescatarian, and vegetarian food patterns in the 31–50-year-old female (**A**) and 31–50-year-old male (**B**) data. Dots represent the outliers.

There was a monotonic decrease in the total animal protein content from the modeled US reference to the vegetarian food patterns, both for males and females. In contrast, there was a monotonic increase in the total plant protein content from the modeled US reference to vegetarian food patterns for both sexes. For the total protein foods, the highest amount was seen in the modeled US reference (5.05 oz eq for females and 7.89 oz eq for males), and the lowest was for the modeled pescatarian pattern (3.51 oz eq for females and 5.51 oz eq for males). Finally, the total amounts of animal protein and plant protein were higher in the modeled male data (Appendix A Figure A3B) compared with the modeled female data (Appendix A Figure A3A).

#### 3.1.5. Healthy Eating Index

**Figure A4:**
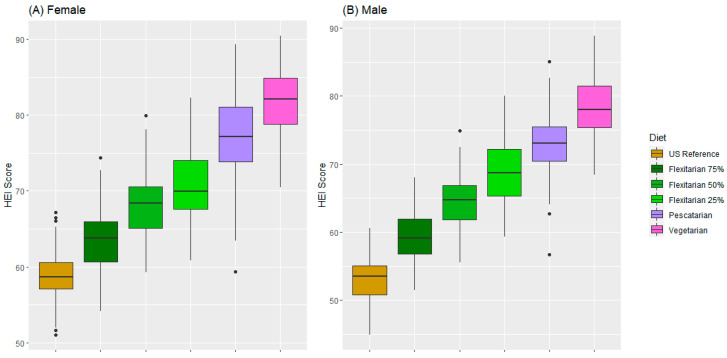
Healthy Eating Index of the different modeled food patterns for (**A**) females and (**B**). Dots represent the outliers.

Table 4 presents the HEI-2015 scores and sub-scores for the modeled food patterns for females and Table 5 presents the HEI-2015 scores and sub-scores for modeled food patterns for males. Corresponding box plots are presented in Appendix A.3 and Appendix A Figure A4.

In general, the modeled food patterns for females had statistically significantly higher HEI-2015 scores than those for males (*p*-value = 7 × 10^−13^, results not shown). The modeled male data obtained overall higher scores of the sub-categories that refer to nutrients (i.e., added sugars and sodium, saturated fats) rather than food groups (i.e., total fruit, whole fruit, total vegetable, greens and beans, whole grains, dairy, total protein foods, and seafood and plant proteins). For both sexes, the highest HEI-2015 scores were obtained for those following a modeled vegetarian food pattern (82 for females and 78 for males), followed by a modeled pescatarian food pattern (77 for females and 73 for males). The lowest HEI-2015 score for both sexes was obtained in the modeled US reference pattern, specifically 59 for females and 53 for males. The total HEI-2015 scores were significantly different between the groups for both sexes (*p* < 0.001). Moreover, as the intake of animal protein decreased, the HEI score increased. In terms of the individual components, a higher HEI-2015 score for female vegetarians was explained by the closer adherence to the guidelines for total and whole fruit, total vegetables, whole grains, the fatty acids ratio, seafood and plant proteins, refined grains, sodium, and saturated fats. Similar conclusions can be drawn from Table 4 and Table 5, where all the different patterns for both female and male data are presented, respectively.

## 4. Discussion

Many segments of the population strive to improve their diets by adopting specific eating patterns. The nutritional quality of some of these patterns may also have relevance for public health policies. The NHANES database s used to assess the nutritional status of adults and children in the United States based on one or two 24 h recalls. Whether a given person follows a specific pattern based on two days of 24 h dietary recalls is, however, hard to say with any certainty. We, therefore, elected to model food patterns using a novel technique based on assembling the observed meals into simulated food patterns. As long as the definitions and the rules for building the modeled food patterns are clear, the method can be applied to new and emerging dietary patterns, including those with a high content of plant proteins rather than animal proteins. In order to ensure realistic food choices, we leveraged observed meals for adults aged 31–50 years in the NHANES 2017–2018 database. The included meals were breakfasts, lunches, dinners, and snacks. The meals were sampled to generate 7-day meal plans that were assigned into a food pattern (e.g., flexitarian, pescatarian, and vegetarian) following specific rules.

MILP ensured that the modeled food patterns (e.g., flexitarian, pescatarian, and vegetarian) did not excessively deviate from the typical American diet (US reference or omnivorous). Our study was the first to introduce MILP in diet modeling to the best of our knowledge; this methodological step greatly enhances the possibilities of mathematical optimization as a tool to model diets. Typically, linear modeling or data envelopment analysis is used to optimize diets; however, these methods only allow for continuous variables and are limited in their capability of modeling logical constraints. By contrast, integer-valued variables can be used to model categorical data, for instance, for categorizing meals or foods as appropriate for a specific meal occasion or inclusion in a given dietary pattern. This greatly improves the acceptability of the generated diets and, therefore, represents major methodological progress [15,23].

Along with other researchers [24], we identified and described some dietary patterns as flexitarian, vegetarian (lacto-ovo-vegetarian), and pescatarian. The current literature is unclear on the precise definition of flexitarian [25]. Our definition of flexitarian dietary patterns was based on a progressive restriction of daily calories from animal foods. Furthermore, the number of individuals following some of the studied diets in the NHANES 2017–2018 database is likely to be relatively small [24]. Past studies done with NHANES data from 2007–2010 indicated that around 2.1% of Americans self-identified as vegetarians; of this 2.1%, only 3% reported consuming no animal protein foods (vegans) [26]. Finding a sufficient number of individuals in the NHANES 2017–2018 database who followed a given dietary pattern presented a particular challenge, which could be circumvented with our computational approach.

Compliance with the Dietary Guidelines for Americans is typically measured using the HEI, which is a measure of diet quality that takes both nutrients and food groups into account [22]. The HEI-2015 favors seafood and plant proteins, dairy, and mono- and polyunsaturated fats but penalizes saturated fats, added sugars, and sodium. In general. HEI scores > 80 indicate a “good” diet, scores ranging from 51 to 80 reflect a diet that “needs improvement,” and HEI scores < 51 imply a “poor” diet [27]. Analyses of the NHANES 2017–2018 data showed that the average HEI-2015 score for adults was 58/100, with scores of 56/100 for males and 60/100 for females [16,22]. In the present study, the US reference (omnivore) food pattern that was generated using MILP had an average HEI-2015 score of 56, with scores of 53/100 for males and 59/100 for females. Those values were identical to those obtained in the What We Eat in America studies [22].

The present evaluations of modeled food patterns support the conclusions of a recent review based on 12 studies, where [28] showed that (ovo-lacto) vegetarians had higher HEI-2010 scores compared with omnivores (72.81 vs. 56.44, *p* < 0.001). In general, a higher diet quality score was obtained by vegetarians or vegans (4.5–16.4 points higher on the HEI-2015) compared with non-vegetarians. Higher HEI-2010 scores were explained by the closer adherence among vegetarians to dietary guidelines for total fruit, whole grains, seafood and plant protein, and sodium. By contrast, non-vegetarians had higher HEI-2010 sub-scores for refined grains and total protein foods [28]. In another study [29], NHANES 2005–2010 1-day recalls were categorized as pescatarian, vegetarian, or omnivore based on the presence/absence of specific foods. The mean HEI-2010 scores were the highest for pescatarian diets (58.76 ± 0.79) and higher (*p* < 0.05) for vegetarian diets (51.89 ± 0.74) than for omnivore diets (48.92 ± 0.33). These differences in diet quality were confirmed when using the alternative HEI.

Analyses of our HEI-2015 sub-scores support these findings, showing in addition that vegetarians also scored higher on vegetables and greens and beans. The modeled pescatarian food patterns scored higher on total fruits, whole fruits, and dairy. To our knowledge, only one study [30] evaluated flexitarian dietary patterns for overall nutritional value; in a healthy German adult population, flexitarian diets had a significantly better diet quality than omnivores but lower than vegans. Diet quality was measured using a modified version of the HEI-2010, namely, the HEI-Flex.

In the present analyses, modeled flexitarian food patterns, which were defined by a reduced content of animal-sourced foods, were associated with higher HEI-2015 scores. Mean HEI-2015 scores ranged from 57/100 to 71/100 with higher values generally obtained for women as opposed to men. These modeled data demonstrate that a shift toward a plant-based diet with less animal protein was not associated with any reduction in diet quality, at least as measured by the HEI-2015. It should be noted that given the very high consumption of protein in the US (which was incorporated into the MILP model) HEI-2015 values for total protein foods were already at the ceiling and no measurable improvements could take place. The seafood and plant protein score increased from 4 to the maximum of 5 points.

Modeling methodologies were used in the past to evaluate the quality of plant-based diets. For example, recent modeling studies substituted animal meat with meat alternatives and/or non-dairy plant-based beverages [28,31,32,33,34,35]. Other studies began to explore the relationship between diet quality scores and the impact of a given dietary pattern on the environment [36,37,38,39]. However, the present modeling approach offers new insight into how different types of diets with varying levels of animal-based and plant-based protein foods may impact our food consumption and diet quality. In particular, we explored the nutritional impact of modeled food patterns characterized by a progressive restriction of animal foods, including animal protein (the flexitarian patterns at different levels). This issue will become progressively more important as plant-based diets are adopted by more segments of the US and European populations. The HEI-2015 scoring system already recognizes that plant-based diets are rich in nutrient-dense foods, such as whole grains, fruits, vegetables, nuts, and legumes, which are good sources of unsaturated fats; dietary fibers; and some vitamins, minerals, and phytonutrients [22].

### Strengths and Limitations

Nutritional analysis of dietary patterns is often limited by the availability of data. The present method overcomes the problem of data scarcity by creating theoretical food patterns that are based on observed data for a representative sample of the US population. Being able to generate multiple days of a given dietary pattern in optimization models is novel. This approach resolves some past methodological challenges. The present MILP model used existing meals of the US population rather than dietary guidelines to generate sets of dietary patterns that followed real-life behaviors. The algorithmic generation of food patterns also made it extremely resource-efficient compared with manually generated food patterns.

To our knowledge, this was the first study to model the nutritional impact of a progressive reduction in animal-sourced foods at a population level, using real meals rather than individual foods to recreate realistic diets. Other studies tried to model the flexitarian dietary pattern by replacing 50% of each animal-based food consumed with an equal amount of plant-based food [33,34]. By contrast, using our method, the total consumption of animal-based foods over the entire diet was reduced by 50%. Our use of MILP allowed us to preserve the total meal and ensure that each modeled day contained exactly one each of breakfast, lunch, dinner, and snack. This allowed us to control the total consumption of animal foods at daily or weekly levels while using actual meals.

This present methodology allowed the flexibility to model any type of diet that can be defined by a set of food groups or nutrient rules. Therefore, this approach can be applied to different countries and dietary patterns when data is available. However, there are limitations. First, we need to have enough qualifying meals in NHANES to allow for the creation of the desired number of simulated weeks of diet. Second, people do not always follow rules when selecting their foods for a given day. The result is that the modeled food patterns tend to mimic the “average” diet, as defined by the usual intakes, rather than capturing the full diversity of meals that would be eaten by the actual population. Another limitation is that eating preferences are not accounted for. The optimization will select whatever meals best meet the constraints, regardless of whether those meals are commonly consumed or contain commonly consumed foods.

In addition, looking at indicators of overall diet quality may not be sufficient to determine the nutritional adequacy of flexitarian, pescatarian, or vegetarian diets. Comparison with the HEI, which comprises only 13 components that are mostly food groups, is useful to assess the adherence of the modeled diets to the dietary guidelines. However, diet quality cannot be assessed exclusively using the HEI score and further research is necessary to look at nutrient intakes. In addition to nutrient status, the risk of deficiencies and long-term impact on health outcomes of these diets cannot be assessed by using only simulated diets. Finally, special attention needs to be given to the different sources of protein and the impact that shifting from animal proteins to plant proteins might have on the nutrient intake and diet quality of certain segments of the population.

Further limitations of this study include the self-reported dietary intake data from NHANES, which is memory-dependent and imposes a change in habitual intake. Other types of diets, such as vegan, were not included in the analysis, as the lack of qualifying meals in the dataset made them impracticable to simulate (see Table 2). Furthermore, as only results of specific age ranges and only from the US were reported, generalized conclusions cannot be drawn. Lastly, 100 weekly diets were simulated as a proof of concept, and future applications might need a sample size calculation.

## 5. Conclusions

A novel computational approach was applied to model a range of food patterns characterized by a reduction in animal protein. The MILP model used meals reported in NHANES 2017–2018 as building blocks of the simulated food patterns generated subject to several nutritional constraints. The modeled reduction in animal protein consumption led to higher HEI-2015 scores. The highest HEI-2015 scores were obtained for the modeled vegetarian food pattern, followed by the modeled pescatarian pattern. The modeled flexitarian pattern in which total animal protein was reduced to 25% seems to be a great alternative for those who choose to consume fewer animal protein sources and can also act as a transition from the omnivore toward the fully plant-based dietary patterns. This food pattern modeling method based on existing meal-level data can be applied to different age groups, populations, or food cultures for greater diversity. With the growing consumer trend of plant-based meat alternatives, the impact of such alternatives on diet quality needs to be investigated.

## Figures and Tables

**Table 1:** Description of alternative modeled food patterns in optimization analysis.

Food Patterns	Meal Inclusion Criteria for Each Modeled Food Pattern
US reference or omnivore	Meals can contain foods of both animal and plant origin, i.e., omnivore. Excluded food patterns components *: none.
75% flexitarian (Flexi75)	Meals can contain foods of both plant origin and animal origin but meals containing animal-sourced foods are limited to 75%. Excluded food patterns components: none.
50% flexitarian (Flexi50)	Meals can contain foods of both plant origin and animal origin but meals containing animal-sourced foods are limited to 50%. Excluded food patterns components: none.
25% flexitarian (Flexi25)	Meals can contain foods of both plant origin and animal origin but meals containing animal-sourced foods are limited to 25% Excluded food patterns components: none.
Pescatarian	Meals cannot contain meat, poultry, cured meat, or organ meat. Excluded food pattern components: total protein foods (incl. meat, poultry, cured meat, organ meat).
Vegetarian or ovo-lacto-vegetarian	Meals cannot contain meat, poultry, cured meat, organ meat, or seafood. Excluded food pattern components: total animal protein foods (incl. meat, poultry, cured meat, organ meat, seafood low in *n*-3, seafood high in *n*-3).

* Food Patterns Equivalents Database (FPED): see Appendix A.1 for detailed information on the different FPED components. The total protein foods (in ounces equivalent) included meat, poultry, organ meat, cured meat, seafood high in *n*-3, seafood low in *n*-3, eggs, soy products, and nuts and seeds. The total protein foods did not include legumes (including beans, peas, and lentils); thus, legumes were considered a separate food pattern component. The total protein foods did not include total dairy (including milk, yogurt, and cheese, in cup equivalents); thus, a proportion of total animal protein intake was considered separately. These terms are used as defined here throughout this paper.

**Table 2:** Classification and counts of the adult meals reported in NHANES into occasions and food patterns.

	Total Meals	Breakfast	Lunch	Dinner	Snack
Omnivore	28,802	7527	6963	8395	5917
Flexitarian	28,802	7527	6963	8395	5917
Pescatarian	15,305	5663	2269	2183	5190
Vegetarian	13,956	5587	1818	1489	5062
Vegan	1842	511	365	206	760

**Table 3:** List of optimization constraints (lower and upper limits of the allowed range) for the US reference modeled food pattern (omnivore). Constraints were applied daily (d) or weekly (w).

Nutrient and Food Pattern Component (Unit, Time Span)	Male		Female	
	Lower	Upper	Lower	Upper
Total fruit (cups, d)	0.3	1.3	0.4	1.3
Total vegetables, excl. beans and peas (cups, d)	1.3	2.1	1.0	1.8
Beans and peas (cups, w)	0.0	1.4	0.0	0.7
Total grains (oz, d)	6.1	9.3	4.3	6.6
Total protein foods, incl. beans and peas (oz, d)	6.8	10.2	4.4	6.8
Total meat, poultry, seafood, and eggs (oz, d)	5.4	8.5	3.3	5.6
Total soy, nuts, and seeds (oz, w)	3.5	14.0	2.8	10.5
Total protein from legumes (beans and peas; oz, w)	1.4	5.6	0.7	3.5
Total dairy (cups, d)	1.1	2.4	0.9	1.9
Oils (grams, d)	18.6	32.3	13.4	23.2
Solid fats (grams, d)	32.9	56.7	22.9	38.6
Sodium (mg, d)	2734	3681	1900	3681
Energy from added sugars (kcal)	216	336	180	270
Energy (kcal)	2096	2951	1490	2069
Energy from added sugars (% daily energy)	9	14	10	15

**Table 4:** Healthy Eating Index 2015 scores for the modeled female data.

Food Pattern Components of the Healthy Eating Index 2015 for Modeled Female Data (Max Points)	US Reference	Flexitarian 75%	Flexitarian 50%	Flexitarian 25%	Pescatarian	Vegetarian	*p*_anova_
Total fruit (5)	2.9 ± 0.3	3.0 ± 0.4	3.0 ± 0.3	4.3 ± 0.6	4.2 ± 0.7	4.1 ± 0.5	<0.001
Whole fruit (5)	4.3 ± 0.7	4.4 ± 0.7	4.5 ± 0.7	4.8 ± 0.6	5.0 ± 0.2	5.0 ± 0.2	<0.001
Total vegetable (5)	3.6 ± 0.2	3.7 ± 0.2	3.8 ± 0.3	4.4 ± 0.4	4.0 ± 0.6	4.1 ± 0.5	<0.001
Greens and beans (5)	3 ± 1	3 ± 1	3 ± 1	3 ± 2	2 ± 2	3 ± 2	<0.001
Whole grains (10)	4 ± 2	4 ± 2	4 ± 2	6 ± 2	7 ± 2	7 ± 2	<0.001
Dairy (10)	5.8 ± 0.5	3.6 ± 0.4	2.0 ± 0.5	1.3 ± 0.5	6.0 ± 0.9	6.6 ± 0.7	<0.001
Total protein foods (5)	5 ± 0	5 ± 0	5 ± 0	5 ± 0	5 ± 0	5 ± 0	1
Seafood and plant proteins (5)	4.0 ± 0.8	4.6 ± 0.5	5 ± 0	5 ± 0	5 ± 0	5 ± 0	<0.001
Fatty acids ratio (10)	2.9 ± 0.7	5 ± 1	8 ± 1	9.9 ± 0.2	9.7 ± 0.5	9.9 ± 0.3	<0.001
Refined grains (10)	7 ± 1	7 ± 1	7 ± 1	5 ± 2	6 ± 2	8 ± 1	<0.001
Sodium (10)	4.7 ± 0.9	6 ± 1	6 ± 1	6 ± 2	7 ± 1	7.1 ± 0.9	<0.001
Added sugars (10)	6.9 ± 0.3	6.8 ± 0.3	6.7 ± 0.3	6.5 ± 0.3	6.6 ± 0.6	6.7 ± 0.3	<0.001
Saturated fats (10)	5.2 ± 0.7	8.3 ± 0.9	10.0 ± 0.1	10 ± 0	10 ± 0	10 ± 0	<0.001
Overall HEI score (100)	59 ± 3	64 ± 4	68 ± 4	71 ± 5	77 ± 6	82 ± 4	<0.001

**Table 5:** Healthy Eating Index 2015 scores for the modeled male data.

Food Pattern Components of the Healthy Eating Index 2015 for Modeled Male Data (Max Points)	US Reference	Flexitarian 75%	Flexitarian 50%	Flexitarian 25%	Pescatarian	Vegetarian	*p*_anova_
Total fruit (5)	2.0 ± 0.3	2.2 ± 0.3	2.3 ± 0.3	3.5 ± 0.7	3.5 ± 0.8	3.3 ± 0.5	<0.001
Whole fruit (5)	3.2 ± 0.7	3.0 ± 0.7	3.6 ± 0.8	4.5 ± 0.8	4.6 ± 0.8	4.5 ± 0.8	<0.001
Total vegetable (5)	3.4 ± 0.2	3.5 ± 0.2	3.7 ± 0.2	4.1 ± 0.4	3.7 ± 0.4	4.1 ± 0.5	<0.001
Greens and beans (5)	3 ± 1	3 ± 1	4 ± 1	4 ± 1	3 ± 1	4 ± 1	<0.001
Whole grains (10)	3 ± 1	4 ± 2	4 ± 2	5 ± 2	6 ± 2	8 ± 2	<0.001
Dairy (10)	5.3 ± 0.5	3.8 ± 0.5	2.1 ± 0.4	1.4 ± 0.4	5.3 ± 0.9	5.6 ± 0.7	<0.001
Total protein foods (5)	5 ± 0	5 ± 0	5 ± 0	5 ± 0	5 ± 0	5 ± 0	1
Seafood and plant proteins (5)	4.0 ± 0.9	4.8 ± 0.4	5 ± 0	5 ± 0	5 ± 0	5 ± 0	<0.001
Fatty acids ratio (10)	3.0 ± 0.8	4.7 ± 0.9	8 ± 1	9.9 ± 0.4	9 ± 1	10 ± 0.4	<0.001
Refined grains (10)	6 ± 1	6 ± 1	6 ± 1	4 ± 2	5 ± 2	6 ± 2	<0.001
Sodium (10)	4 ± 1	5 ± 1	5 ± 1	5 ± 1	6 ± 1	6 ± 1	<0.001
Added sugars (10)	7.4 ± 0.3	7.3 ± 0.3	7.2 ± 0.3	7.2 ± 0.3	7.5 ± 0.6	7.4 ± 0.3	<0.001
Saturated fats (10)	4.0 ± 0.9	7.2 ± 0.7	9.8 ± 0.3	10 ± 0	10.0 ± 0.1	10 ± 0	<0.001
Overall HEI score (100)	53 ± 3	59 ± 4	65 ± 4	69 ± 4	73 ± 5	78 ± 5	<0.001

## Data Availability

Data generated in this study is available in the Supplementary Materials. NHANES data is publicly available from the Centers for Disease Control and Prevention’s National Center for Health Statistics: https://wwwn.cdc.gov/nchs/nhanes/continuousnhanes/default.aspx?BeginYear=2017 (accessed on 28 April 2023).

## Supplementary Materials

The following supporting information can be downloaded from https://www.mdpi.com/article/10.3390/nu15112572/s1: weekly average of all nutritional components for the 100 simulated meal plans per modeled food pattern, grouped by sex.

## Appendix A

### Appendix A.1. Food Patterns Equivalents Database

**Table A1:** Protein FPED components used to model the omnivore, flexitarian, pescatarian, and vegetarian food patterns. Adapted from https://www.ars.usda.gov/ARSUserFiles/80400530/pdf/fped/FPED_1718.pdf (accessed on 28 April 2023).

FPED Component and SAS Variable Name	Foods and Units
Total protein foods (PF_TOTAL)	Total meat, poultry, organ meat, cured meat, seafood, eggs, soy, and nuts and seeds; excludes legumes (oz. eq.)
Total meat, poultry, and seafood (PF_MPS_TOTAL)	Total of meat, poultry, seafood, organ meat, and cured meat (oz. eq.)
Meat (PF_MEAT)	Beef, veal, pork, lamb, and game meat; excludes organ meat and cured meat (oz. eq.)
Cured meat (PF_CUREDMEAT)	Frankfurters, sausages, corned beef, cured ham, and luncheon meat that are made from beef, pork, or poultry (oz. eq.)
Organ meat (PF_ORGAN)	Organ meat from beef, veal, pork, lamb, game, and poultry (oz. eq.)
Poultry (PF_POULT)	Chicken, turkey, Cornish hens, duck, goose, quail, and pheasant (game birds); excludes organ meat and cured meat (oz. eq.)
Seafood high in *n*-3 fatty acids (PF_SEAFD_HI)	Seafood (finfish, shellfish, and other seafood) high in *n*-3 fatty acids (oz. eq.)
Seafood Low in *n*-3 fatty acids (PF_SEAFD_LOW)	Seafood (finfish, shellfish, and other seafood) low in *n*-3 fatty acids (oz. eq.)
Eggs (PF_EGGS)	Eggs (chicken, duck, goose, and quail) and egg substitutes (oz. eq.)
Soy products (PF_SOY)	Soy products, excluding calcium-fortified soy milk (soymilk) and products made with raw (green) soybean (oz. eq.)
Nuts and seeds (PF_NUTSDS)	Peanuts, tree nuts, and seeds; excludes coconut (oz. eq.)
Beans, peas, and lentils (PF_LEGUMES)	Beans, peas, and lentils (legumes) computed as protein foods (oz. eq.)
Total dairy (D_TOTAL)	Total milk, yogurt, cheese, and whey; for some foods, the total dairy values could be higher than the sum of D_MILK, D_YOGURT, and D_CHEESE because the miscellaneous dairy component composed of whey is not included in FPED as a separate variable (cup eq.)
Milk (D_MILK)	Fluid milk, buttermilk, evaporated milk, dry milk, and calcium-fortified soy milk (soymilk) (cup eq.)
Yogurt (D_YOGURT)	Yogurt (cup eq.)
Cheese (D_CHEESE)	Cheeses (cup eq.)

### Appendix A.2. Optimization Constraints for the Modeled Flexi75, Flexi50, Flex25, Pescatarian, and Vegetarian Patterns

**Table A2:** List of optimization constraints (lower and upper limits of the allowed range) for the Flexi75 modeled food pattern. Constraints were applied daily (d) or weekly (w).

Food Pattern Component (Unit, Time Span)	Male		Female	
	Lower	Upper	Lower	Upper
Total fruit (cups, d)	0.3	1.3	0.4	1.3
Total vegetables, excl. beans and peas (cups, d)	1.3	2.1	1.0	1.8
Beans and peas (cups, w)	0.0	1.75	0.0	0.9
Total grains (oz, d)	6.1	9.3	4.3	6.6
Total protein foods, incl. beans and peas (oz, d)	6.8	10.2	4.4	6.8
Total meat, poultry, seafood, and eggs (oz, d)	3.3	6.4	1.9	4.2
Total soy, nuts, and seeds (oz, w)	4.4	17.5	3.5	13
Total protein from legumes (beans and peas; oz, w)	1.75	7	0.88	4.38
Total dairy (cups, d)	0.5	1.8	0.43	1.43
Oils (grams, d)	18.6	32.3	13.4	23.2
Solid fats (grams, d)	18	32.3	13.25	29.95
Sodium (mg, d)	2734	3681	1900	3681
Energy from added sugars (kcal)	216	336	180	270
Energy (kcal)	2096	2951	1490	2069
Energy from added sugars (% daily energy)	9	14	10	15

**Table A3:** List of optimization constraints (lower and upper limits of the allowed range) for the Flexi50 modeled food pattern. Constraints were applied daily (d) or weekly (w).

Food Pattern Component (Unit, Time Span)	Male		Female	
	Lower	Upper	Lower	Upper
Total fruit (cups, d)	0.3	1.3	0.4	1.3
Total vegetables, excl. beans and peas (cups, d)	1.3	2.1	1.0	1.8
Beans and peas (cups, w)	0.0	2.1	0.0	1
Total grains (oz, d)	6.1	9.3	4.3	6.6
Total protein foods, incl. beans and peas (oz, d)	6.8	10.2	4.4	6.8
Total meat, poultry, seafood, and eggs (oz, d)	1.2	4.3	0.5	2.8
Total soy, nuts, and seeds (oz, w)	5.25	21	4.2	16
Total protein from legumes (beans and peas; oz, w)	2.1	8.4	1.1	5.25
Total dairy (cups, d)	0	1.2	0	0.95
Oils (grams, d)	18.6	32.3	13.4	23.2
Solid fats (grams, d)	4	28.35	3.6	19.3
Sodium (mg, d)	2734	3681	1900	3681
Energy from added sugars (kcal)	216	336	180	270
Energy (kcal)	2096	2951	1490	2069
Energy from added sugars (% daily energy)	9	14	10	15

**Table A4:** List of optimization constraints (lower and upper limits of the allowed range) for the Flexi25 modeled food pattern. Constraints were applied daily (d) or weekly (w).

Food Pattern Component (Unit, Time Span)	Male		Female	
	Lower	Upper	Lower	Upper
Total fruit (cups, d)	0.3	1.3	0.4	1.3
Total vegetables, excl. beans and peas (cups, d)	1.3	2.1	1.0	1.8
Beans and peas (cups, w)	0.0	2.45	0.0	1.2
Total grains (oz, d)	6.1	9.3	4.3	6.6
Total protein foods, incl. beans and peas (oz, d)	6.8	10.2	4.4	6.8
Total meat, poultry, seafood, and eggs (oz, d)	0	2.2	0	1.4
Total soy, nuts, and seeds (oz, w)	6.1	23	4.9	18
Total protein from legumes (beans and peas; oz, w)	2.45	9.8	1.2	6.13
Total dairy (cups, d)	0	0.6	0	0.48
Oils (grams, d)	18.6	32.3	13.4	23.2
Solid fats (grams, d)	0	14.18	0	9.65
Sodium (mg, d)	2734	3681	1900	3681
Energy from added sugars (kcal)	216	336	180	270
Energy (kcal)	2096	2951	1490	2069
Energy from added sugars (% daily energy)	9	14	10	15

**Table A5:** List of optimization constraints (lower and upper limits of the allowed range) for the modeled pescatarian food pattern. Constraints were applied daily (d) or weekly (w).

Food Pattern Component (Unit, Time Span)	Male		Female	
	Lower	Upper	Lower	Upper
Total fruit (cups, d)	0.3	1.3	0.4	1.3
Total vegetables, excl. beans and peas (cups, d)	1.3	2.1	1.0	1.8
Beans and peas (cups, w)	0.0	2.8	0.0	1.4
Total grains (oz, d)	6.1	9.3	4.3	6.6
Total protein foods, incl. beans and peas (oz, d)	6.8	10.2	4.4	6.8
Total meat, poultry, seafood, and eggs (oz, d)	0.2	1.8	0.2	1.3
Total seafood (oz, w)	1.4	6.3	1.4	4.9
Total soy, nuts, and seeds (oz, w)	7	28	5.6	21
Total protein from legumes (beans and peas; oz, w)	2.8	11.2	1.4	7
Total dairy (cups, d)	1.1	2.4	0.9	1.9
Oils (grams, d)	18.6	32.3	13.4	23.2
Solid fats (grams, d)	0	14.175	0	9.65
Sodium (mg, d)	2734	3681	1900	3681
Energy from added sugars (kcal)	216	336	180	270
Energy (kcal)	2096	2951	1490	2069
Energy from added sugars (% daily energy)	9	14	10	15

**Table A6:** List of optimization constraints (lower and upper limits of the allowed range) for the modeled vegetarian food pattern. Constraints were applied daily (d) or weekly (w).

Food Pattern Component (Unit, Time Span)	Male		Female	
	Lower	Upper	Lower	Upper
Total fruit (cups, d)	0.3	1.3	0.4	1.3
Total vegetables, excl. beans and peas (cups, d)	1.3	2.1	1.0	1.8
Beans and peas (cups, w)	0.0	2.8	0.0	1.4
Total grains (oz, d)	6.1	9.3	4.3	6.6
Total protein foods, incl. beans and peas (oz, d)	6.8	10.2	4.4	6.8
Total meat, poultry, seafood, and eggs (oz, d)	0	0.9	0	0.6
Total soy, nuts, and seeds (oz, w)	7	28	5.6	21
Total protein from legumes (beans and peas; oz, w)	2.8	11.2	1.4	7
Total dairy (cups, d)	1.1	2.4	0.9	1.9
Oils (grams, d)	18.6	32.3	13.4	23.2
Solid fats (grams, d)	0	14.18	0	9.65
Sodium (mg, d)	2734	3681	1900	3681
Energy from added sugars (kcal)	216	336	180	270
Energy (kcal)	2096	2951	1490	2069
Energy from added sugars (% daily energy)	9	14	10	15
